# Assessing the Social and Environmental Impact of Healthcare Technologies: Towards an Extended Social Return on Investment

**DOI:** 10.3390/ijerph20065224

**Published:** 2023-03-22

**Authors:** Maria Pinelli, Stefania Manetti, Emanuele Lettieri

**Affiliations:** Department of Management, Economics and Industrial Engineering, Politecnico di Milano, Via Lambruschini 4/B, 20156 Milan, Italy

**Keywords:** SROI, technology, disability, exoskeleton

## Abstract

Stroke is the third leading cause of death and disability overall worldwide. Upper limb impairment is a common consequence for stroke survivors, having negative impact on their quality of life. Robotic rehabilitation, through repetitive and monitored movements, can improve their status. Developed by a team of researchers at Politecnico di Milano, AGREE is an exoskeleton for upper limb rehabilitation at the stage gate between translational research and clinical validation. Since the cost of this device is particularly high, the present study aimed to provide a framework for assessing its value. The Social Return on Investment (SROI) method, able to grasp the economic, social and environmental impact of an activity, was applied, using expert opinions of a pool of clinical engineers and healthcare professionals from different Italian hospitals to obtain information. Environmental impacts were estimated through Life Cycle Assessment in terms of CO_2_ emissions and incorporated in the analysis. Considering a 5-year period, the SROI for a single exoskeleton was 3.75:1, and the SROI for the number of exoskeletons projected to be sold was 2.868:1, thus resulting largely in value for money. This study provides a model for combining economic, social and environmental outcomes that, besides contributing to theory, could be useful for decision-making.

## 1. Introduction

As defined by the World Health Organization (WHO) in 1970, “stroke is rapidly developing clinical signs of focal (or global) disturbance of cerebral function, with symptoms lasting 24 h or longer, or leading to death, with no apparent cause other than of vascular origin” [1]. 

The latest Global Burden of Disease (GBD) 2019 identified stroke as the second leading global source of death, and the third leading global source of death and disability together, intended as disability-adjusted life years (DALYs) lost [2].

People who survive a stroke often incur disabilities [3], which can include physical, cognitive and communication problems. About a quarter of strokes arise in people under the age of sixty-five [4], disproving the view that strokes are only an issue for ‘older people’.

Usually, people who experience a moderate to severe stroke are first hospitalised and then discharged home or to a rehabilitation centre to continue their rehabilitation. 

In particular, it has been found that continuing rehabilitation even afterwards is beneficial to maintain functional gains and facilitate adjustment [5]. 

The presence of community services, family and friends [6], alongside the existence of a broader social network after discharge from hospital, improves the success rate for post-stroke patients.

Common problems of stroke patients are an impaired capacity of walking and lower confidence in usual activities, such as using public transportation and driving [7]. 

Moreover, upper limb impairment is one of the most recurring outcomes in patients soon after a stroke, prevalent in 70–80% of patients [8]. 

Since upper limb impairment after stroke has a significant impact on the ability of the affected person to perform an activity, it negatively affects their quality of life [9]. For this reason, there is a particular need for developing training strategies tailored to the needs and desires of stroke survivors [10]. Considering the frequent recurrence of upper limb impairment after stroke, this has been an important topic of discussion for years, and, in 2012, it was listed by The Lancet as one of the top ten most frequently raised issues among both patients and professionals working with stroke patients [11].

Nevertheless, not all paresis are equally severe, and depending on the severity, the upper limb functionality varies greatly. Hemiparesis is the most important impairment leading to loss of upper limb function, and the severity of hemiparesis correlates with the ability to perform a movement or an action [12]. However, the ability to perform tasks using the upper limb is also affected by other factors such as somatosensory deficiencies [13], shoulder pain [14] or intellectual deficits [15].

In recent years, the scientific community has shown great interest in the research and development of technologies for upper limb rehabilitation and assistance in performing activities of daily living in the home environment, in cases of chronic residual disability. Studies of functional reorganisation of the central nervous system following injury have shown that early motor activity during rehabilitation training has a significant impact on functional recovery [16].

Certain conditions associated with the use of robotic rehabilitation appear to particularly influence functional outcomes. Indeed, it has been observed that in long-term upper limb treatments, therapy involving high-intensity activity features with repetitive, controlled and goal-directed movements—well suited to the use of robotic devices with controlled tasks—offers a significant improvement in functional recovery compared to conventional treatments [17,18].

The use of robots in rehabilitation allows us not only to maximise the number of repetitions of the same exercise, which is a key element for neural pathway reconstruction, but also to adjust the contribution required from the patients so that they always work at the limit of their remaining motor skills, with an ‘assisted as needed’ approach. Finally, robotic devices used in rehabilitation typically interact with objects or immersive graphic interfaces that help patients maintain high levels of attention and engagement—other critical factors for an effective and rapid functional recovery pathway. The support needed can vary greatly depending on the residual disability of the patient’s upper limb.

In commercial terms, the rehabilitation robotic market has been estimated at USD 226 million in 2021 and is predicted to grow with a Compound Annual Growth Rate (CAGR) of 17.3% from 2022 to 2030 (https://www.grandviewresearch.com/industry-analysis/rehabilitation-robots-market-report, accessed on 10 February 2023), the CAGR being the average yearly increase in revenues between two years. 

On these premises, a team of researchers from the Politecnico di Milano has developed an upper limb rehabilitation robot called AGREE. AGREE is a module-based exoskeleton system for rehabilitation and support of the upper limb, which aims to increase the independence of people with upper limb motor disabilities in everyday activities. The device is at the stage gate between translational research and clinical validation.

Since the production cost of AGREE is particularly high and, as a consequence, so too the purchase cost, the objective of this study was to understand the value that this technology might provide from a societal perspective. In other words, the aim of the authors was to investigate whether this technology represented value for money [19].

Over the years, various models have been used to evaluate health care innovations, such as cost-effectiveness analysis, cost-utility analysis or cost–benefit analysis. 

The *cost-effectiveness analysis* evaluates the costs and outcomes of two technologies compared to each other, by assessing the respective cost of gaining a unit of a health outcome. The basic method for calculating cost-effectiveness is based on comparing years of life lost as the natural unit for measuring the effect of the interventions [20]. A more accurate calculation can be performed by considering disability-adjusted life years (DALYs), which stands for the loss of one year in perfect health (https://www.who.int/data/gho/indicator-metadata-registry/imr-details/158#:~:text=Definition%3A-,One%20DALY%20represents%20the%20loss%20of%20the%20equivalent%20of%20one,health%20condition%20in%20a%20population, accessed on 10 February 2023). Consequently, an increase in DALYs measures the benefits associated with a public health intervention. 

In *cost-utility analysis*, instead, Quality Adjusted Life Years (QALYs), which merge quantity and quality of life [21], are used as the reference unit of measurement. Using QALYs, any potential health intervention can be measured by the extension of each patient’s life expectancy while improving the quality of each year lived.

Finally, *cost–benefit analysis* is an economic evaluation that compares the benefits and losses of an intervention with the costs associated with it [22]. Although this method could be considered fair and accurate, it has been criticized for taking into account only the perspective of one actor and not the totality of stakeholders involved in the intervention [23].

In the last thirty years, the interest in cross-disciplinary frameworks, such as Health Technology Assessment (HTA), has increased. The final aim of this method is to select the most promising biomedical technologies, which can reach patients and society in a timelier fashion. HTA is the systematic assessment of the costs and multidimensional consequences of a medical technology [24,25,26,27]. Recently, there has been an increasing attention to achieve a broader view in HTA by enlarging the scope of analysis and including social, ethical, safety-related or environmental parameters of benefit [25,28]. These criteria are relevant when supporting implementation and adoption of promising technologies into clinical practice.

Another methodology for assessing the value of health technologies has gained acceptance: the Social Return on Investment (SROI). Unlike the cost–benefit analysis, the SROI takes into account different kinds of outcomes, associated with the triple bottom line: economic, social and environmental [29,30]. There is growing interest in assessing the societal impact of public health interventions, but more research is needed to expand this field [31,32].

Considering that multiple stakeholders are involved in the rehabilitation of stroke patients and that costs and outcomes cannot always be monetised, the authors applied the SROI method to build a model to assess the value of the AGREE exoskeleton from a societal perspective.

## 2. Materials and Methods

To address the objective of the research, the authors applied the SROI methodology to assess the value of the AGREE exoskeleton. The model, in particular, aims at solving the failure of the traditional analytical methods, which fall short of accurately identifying all positive and negative externalities.

For the proposed objective, SROI has been used as baseline methodology; environmental impacts have been accounted for through the Life-Cycle Assessment (LCA).

### 2.1. SROI Methodology

As already described, the SROI methodology is an evolution of the cost–benefit analysis, entailing a larger definition of impact.

SROI analysis is based on seven principles [33]: *i. Involve stakeholders; ii. Understand what changes, iii. Value the things that matter, iv. Only include what is material, v. Do not over-claim, vi. Be transparent, vii. Verify the results*.

There are two different types of SROI [34]:

- *Evaluative*, which is carried out ex post retrospectively and based on actual results that have already occurred.
- *Forecast*, which predicts the amount of social value that will be generated if the activities achieve the expected results.

The SROI method is based on the evaluation of an organisation’s activities using an input–output-outcome model with the full involvement of key stakeholders [35]. The six steps required to conduct a SROI analysis are the following: *Establishing scope and identifying stakeholders, Mapping outcomes, Evidencing outcomes and giving them a value, Establishing impact, Calculating the SROI, and Reporting, using and embedding*.

Therefore, it is first important to define the components of the analysis, in terms of objectives, background, time horizon and type of analysis. In this study, the social impact measurement of the AGREE exoskeleton was conducted with a time horizon of 5 years. The useful life of biomedical devices such as AGREE is generally considered to be between 5 and 10 years. It is hoped that AGREE will function for 10 years; however, for the purposes of the economic estimates, it was decided to be prudent and consider a useful life of 5 years. Moreover, SROI estimations are, in general, carried out considering a time horizon between 1 and 5 years [36], so this is coherent with the choice of the authors. 

The SROI Analysis of the AGREE project was carried out as a forecast analysis. In fact, since the exoskeleton is not on the market yet, its social impact has been evaluated for the first 5 years after the launch.

Then, it is important to list all the stakeholders affected by the analysed activity, to understand the changes perceived by them and to define how to involve them in order to better understand and estimate their contribution to the activity and the experienced outcomes. The identified stakeholders were: patients, hospital, physiotherapists, caregivers and employers.

Involving the stakeholders is crucial to avoid autoreferentiality, subjectivity and double counting of measurements. Unfortunately, it was not possible to involve all the stakeholders identified for this analysis, but expert opinion elicitation was used to engage a pool of senior clinical engineers and healthcare professionals working in different Italian hospital settings. This methodology works to retrieve valuable information from crucial stakeholders and it is suitable when the empirical results are limited or insufficient [37].

Then, for each stakeholder, an input–output-outcome-impact chain was developed, and inputs (both monetized and non-monetized) and outcomes were estimated. In this phase, collecting data was fundamental, and values could be established through interviews with the experts, and, when the evidence was limited, extant literature was also consulted. 

In order to calculate the final social impact in a coherent and rational way, it must be verified if the outcomes calculated in the analysis could be uniquely associated with the activity under consideration. This can be achieved through the appraisal of some factors, with the aim of not overestimating the generated impact. These factors are:

- Attribution: the number of outcomes caused exclusively by the activity under consideration;
- Deadweight: a measure of the number of results that would have been obtained even without the activity;
- Displacement: the measure of how much an outcome has displaced other outcomes;
- Drop-off: measures the deterioration of outcomes over the years.

Finally, if the considered time horizon is more than one year, it is necessary to actualize yearly outcomes to obtain the final SROI. The discount rate that was chosen is the one suggested by the UK HM Treasury’s Green Book, since it is the most used in SROI calculations for public initiatives [34]. The value updated to June 2022 is equal to 3.77%, and this was used for the calculations.

In the end, in every investment decision uncertainty must be considered, so it is useful to conduct a sensitivity analysis. Since the model is based on assumptions, the sensitivity analysis can be carried out by modifying one of them and verifying how the results change according to this.

As a final step, the results of the analysis should be communicated and reported, explaining all the hypotheses taken into account and the followed procedure.

### 2.2. Life Cycle Assessment Methodology

LCA is defined as “a methodology for integrated impact assessment that quantifies the (environmental) impacts associated with the entire life cycle of products” [38]. 

The two main standards for conducting an LCA are UNI EN ISO 14040 (2006, 2020) and UNI EN ISO 14044 (2006, 2018, 2020). This iterative methodology is divided into four main steps:

- Definition of goal and scope: this phase defines the goal and components of the study;
- Inventory analysis: in this phase, the life cycle of a product is studied, and all inputs and outputs are identified;
- Impact assessment: in this phase, the data considered in the inventory analysis are combined with the environmental impact categories;
- Interpretation of results: in this final step, the results of the analysis are explained and clarified.

Although there are not many applications of LCA in SROI in the literature, the authors sought to include the environmental impact using the LCA within the SROI. This approach was then applied to the AGREE case to assess its value for money. 

In order to understand how to comprehend environmental considerations in the SROI computation, the authors included the results of the LCA analysis carried out with Open LCA in the outcomes of the SROI, considering as a positive result the reduction in emissions and as a negative result the rise in emissions [39]. 

The monetization of environmental impacts has been performed through the *Benefit Transfer Method*. This is used when there is little evidence to evaluate non-commercial data, and reference is made to already existing studies on environmental or social benefits [39].

### 2.3. Model Assumptions

Before presenting the results of the SROI analysis for AGREE, it is important to clarify that the model is based on assumptions, which may vary from hospital to hospital. The study was made possible employing expert opinion elicitation with a pool of senior clinical engineers and healthcare professionals working in different Italian hospital settings. The SROI was calculated both for a single exoskeleton and for a number of sold exoskeletons estimated by the authors to be sold in the 5 years.

(1) Physiotherapists can treat only one patient per session, both in the case of traditional therapy and in the case of robotic therapy.
(2) The rehabilitation pathway for a post-stroke patient includes a total of 72 h (divided among different rehabilitation therapies). Of these, 20 h are spent on upper limb rehabilitation with traditional therapy, delivered in sessions of one hour each.
(3) The robotic rehabilitation session, with equal efficacy, could result in a 33% reduction in treatment time, due to the higher exercise intensity. The upper limb rehabilitation still lasts 20 sessions, but 40 min each. The time saved (20 min) could be used for other treatments included in the rehabilitation plan, for example, speech therapy, neuropsychology, and generally better recovery of other anatomical districts.
(4) To consider the outcomes obtained for patients, the authors used the Quality of Life (QoL) parameter. Referring to the study by Golicki and colleagues (2015), the QoL at 1 week after stroke (QoL_1week_), measured by the EQ5D3L method, was 0.584, while the QoL 4 months after stroke (QoL_4months_), also measured through the EQ5D3L method, was 0.694 [40]. Since, according to expert opinion, the QoL of the patients treated with AGREE would be not so different from the QoL of the patients treated with traditional rehabilitation, the authors decided to use the value of QoL_1week_ for taking into account the QoL early after stroke, and they used the value of QoL_4months_ for considering the QoL after rehabilitation.
(5) The technology is used on both hospitalized patients and day-hospital patients. Since the likelihood of these two scenarios was unknown, it was assumed that 50% of the patients would be treated in the day-hospital, while inpatient treatment was assumed for the other 50%.
(6) The rate provided by the National Healthcare System to a facility for performing robotic rehabilitation was set equal to the rate for traditional rehabilitation. This corresponds to a Diagnosis-Related Group (DRG) of 278.00 EUR/day. Then, an increase of 8% was taken into account resulting in a total of 300.08 EUR/day in order to also include complex facilities. This amount covers all rehabilitation costs for a neurological patient, including speech therapy, psychology, neurorehabilitation, lower limb rehabilitation, etc. Of all these therapies, upper limb rehabilitation represents 1/3 of the total therapy time, and consequently, 1/3 of the DRG was considered, i.e., 100 EUR per session.

## 3. Results

The analysis was carried out to have a comprehensive view of the likely impact created by the rehabilitation activity performed thanks to the AGREE exoskeleton, identifying outcomes that could be positive, negative, intended or unintended. The results reflected in this section are subject to the base case assumptions used in the model.

### 3.1. Establishing Scope and Identifying Stakeholders

In the following lines, a brief description of the stakeholders involved and the expected changes caused by the initiative are reported. 

Patients: the patients concerned are those that could be treated with the exoskeleton in the 5 years analysed. In particular, both inpatients and outpatients were considered. The impact generated on them would be undoubtedly positive since they are the direct beneficiaries of AGREE, and their recovery could be faster than with conventional therapy.

Hospital: The impact on the hospitals/clinics that decide to purchase the AGREE exoskeleton would be positive, both in terms of the greater number of patients that could be treated with the new equipment and in terms of the better reputation that the institution could have from the point of view of technological progress.

Physiotherapists: The service offered by the exoskeleton would help them optimize their time, increase the number of patients treated, but also increase their knowledge of robotics, which would open up new career opportunities for them.

Caregivers: Rehabilitation, which increases the patients’ independence and quality of life, would have a positive impact on their families by relieving them of the burden of constant care, giving them more free time and improving their mental health.

Employers: Employers of patients involved in rehabilitation were also considered. Indeed, AGREE would be a faster way for them to get their employees back to work, reduce costs associated with “sick leave,” and increase company productivity.

### 3.2. Mapping Outcomes and Giving Them a Value

In the following lines, a description of the inputs, outputs, outcomes and impact for each stakeholder is provided. 

The inputs represent the stakeholder contribution to the activity that is then transformed into outputs. Outcomes are the short-term consequences of the activity, and impacts, instead, represent the long-term consequences of the project.

#### 3.2.1. Patients

Patients participate in the activity dedicating their time to performing the rehabilitation. The direct output is the patient rehabilitation that allows them to reach an improved QoL. In the long term, the impact on patients would be the participation in social activities they were previously unable to join in. In order to monetize the working hours dedicated to performing the rehabilitation, the authors estimated the time required by patients to perform the therapy with the addition of an average travel time in the city of Milan (https://www.universitynetwork.it/ecco-quanto-tempo-trascorriamo-sui-mezzi-pubblici/, accessed on 10 February 2023) to reach a target hospital where the rehabilitation takes place, i.e., about 40 min for the outward journey and 40 min for the return. In the calculations, we considered this value as applicable both for the inpatients and for the patients treated in day-hospital. Indeed, we had no elements to calculate the time dedicated by each patient to the AGREE therapy, apart from the 40 min of duration of the session. We therefore estimated it using the same timings of the day-hospital patients. The inputs, outputs and outcomes for patients are described in Table 1.

Then, considering 8 working hours per day, the working hours dedicated to performing the rehabilitation were monetized through the average salary in Italy (equal to 21,462.62 EUR/year (https://www.key4biz.it/lo-stipendio-medio-in-italia-e-di-21-46262-euro-lanno-i-settori-in-cui-e-diminuito-di-piu/401462/, accessed on 8 March 2023)).

Considering the exoskeleton being used 7 h per day, 275 working days per year and a total of 20 sessions of 40 min each, the number of patients that could be treated by one exoskeleton per year is equal to 144. This number of patients was considered to make the calculations.

In relation to the outcomes for the patients, as previously anticipated, an increase in QoL after the rehabilitation was considered. The values of QoL_1week_ = 0.584 and QoL_4months_ = 0.694 were considered [40]. Considering the budget constraint method, one person’s income limits the ability to purchase, so the average annual salary of patients (EUR 21,462.62) was used to calculate this proxy. 

So, considering one year of life of a patient, it was calculated the willingness to pay for spending that year with a QoL of 0.584 or 0.694 as the yearly salary multiplied by the difference between the two qualities of life.

The second outcome indicator was calculated as the saved time due to the lower duration of the therapy multiplied by the number of patients and monetized through the value of the DRG of therapy, to highlight the possibility to employ the saved hours for other rehabilitation therapies.

#### 3.2.2. Hospitals

There are two inputs for hospitals:

- The purchase of exoskeletons;
- The exoskeleton energy consumption required for its functioning. The total yearly consumption was estimated at 3656.5 KWh/y, monetized through the Unique National Price (PUN) that, since it is not a stable value, was increased by 2% every year, and the mean of marginal costs of the main Italian providers.

The outcome for the hospitals was represented with the increased number of therapies that they can perform thanks to the usage of AGREE. Since the duration of the single therapy session can be reduced by 33.3%, more patients can be treated in the remaining time. The number of increased sessions, then, can be multiplied by the DRG value of EUR 100.08. The inputs, outputs and outcomes for hospitals are described in Table 2.

#### 3.2.3. Physiotherapists

The input considered for physiotherapists was their workforce, calculated as their net average hourly wage (17 EUR/h) multiplied by the AGREE working hours/day (7 h/day) multiplied by 275 days/year. Here, it was not considered that physiotherapists are always working with the exoskeleton, but that for the 7 h/day in which AGREE works, a physiotherapist assists the patient with it. The direct output is patient rehabilitation, with three different types of outcomes identified:

- More time to be dedicated to new patients: this was calculated considering the time saved for each therapy and multiplied by their net average salary.
- Physiotherapists involved in the AGREE rehabilitation process need to complete about 7 h of training to use the exoskeleton in the proper way. To monetize this outcome, the average gross salary of four physiotherapists has been considered as a reference number correspondent to the cost sustained by a target hospital.

As a long-term impact, the use of AGREE, and of robotic rehabilitation in general, would boost their growth as professionals. The inputs, outputs and outcomes for physiotherapists are described in Table 3.

#### 3.2.4. Caregivers

Caregivers give their support to stroke patients in order to successfully perform the rehabilitation activity. Two inputs were considered for this stakeholder:

- Time lost to support the patient during the rehabilitation activity: the working time spent by caregivers to bring the patient to target the hospital/clinic, to wait for the patient to finish the rehabilitation session and to come back home. For the travel time, it can be considered the one already assessed for the patient input computation. Finally, this input has to be calculated only for the caregivers of the outpatients (50% of the patients, according to the assumptions). To monetize this input, the average salary of a caregiver has been used. Caregivers were considered in the Italian D super level, characterized by family assistants of non-sufficient people, with an average salary of 9.24 EUR/h (https://www.contratticcnl.it/, https://www.lebadanti.it/blog/stipendio-colf-e-badanti-2022-tabelle-dei-minimi-retributivi-e-livelli-dinquadramento/ accessed on 10 February 2023).
- Transportation cost: the transportation costs include the fuel expenditure, computed as the average distance in Milan to reach a target hospital, a fuel price per litre of 1.36 EUR/L and an average consumption of a city car of 1 L/13 km. Additionally, this input was calculated for half of the patients.

The outcome considered for caregivers was the increase in free time once their assisted patient has performed the rehabilitation. Indeed, it was assumed that the patient’s rehabilitation corresponds to higher autonomy and, consequently, to a decreased caregiver burden. 

To calculate this outcome, a relationship was established between disability and assistance. Assuming that a patient with a disability of 100% needs 24 h of assistance, a patient with a less disability will need fewer hours of assistance. 

Making a parallelism with the QoL, a patient with a QoL of 1 (perfect health) corresponds to a disability equal to 0%. Considering the QoL_1week_ = 0.584, the disability of a patient early after stroke corresponds to 41.6%, and so the hours needed by the caregiver are 10/day. Then, considering the QoL_4months_ = 0.694 after rehabilitation, the disability percentage becomes 30.6% and the needed hours 7.34. The saved hours, then, were monetized through their average salary of 9.24 EUR/h. Additionally, this outcome was calculated only for the percentage of outpatients (50%).

The long-term impact would consist of the caregivers’ social and work reintegration, which would be beneficial to them lowering their psychological stress. The inputs, outputs and outcomes for caregivers are described in Table 4.

#### 3.2.5. Employers

For the employers, no input was considered. Regarding the outcome, since an employee in Italy can request a maximum of 6 months of sick leave, it has been assumed that the improvement in the QoL could allow them also to require fewer days of sick leave, proportionally to the improvement in QoL, which is around 19%. For the employers, these represent re-employed hours, and so they are monetized with the average employee salary. The inputs, outputs and outcomes for employers are described in Table 5.

### 3.3. Attribution, Deadweight and Drop-Off

First of all, the deadweight has been estimated particularly making reference to the assumption that traditional rehabilitation is 66.6% effective compared with robotic rehabilitation. This was applied to all the outcomes except the outcome related to the QoL since it was considered equivalent to the one obtained through robotic rehabilitation. Regarding attribution, instead, it corresponds to the responsibility of activities for the outcomes. However, since in the evaluation of the outcomes it was always considered only the value created by the robotic rehabilitation through AGREE, a coefficient of 100% was applied. The drop-off, finally, is related to the duration of the equipment. A diffused method of considering the drop-off is to deduct a fixed percentage for each year of the project duration [41]. In this study, a 20% coefficient was employed, due to the fact that the useful life of AGREE was evaluated, as previously reported, at 5 years. 

### 3.4. Life Cycle Assessment of AGREE

This section reports the results of the environmental impact assessment of AGREE. Being AGREE in development, the main difficulty in this evaluation was the availability of reliable data.

The authors, first of all, mapped all the lifecycle processes of AGREE, including the phases from development use, disposal and recycling and performed an inventory analysis describing all the flows associated with the different processes. The assembly and disposal phases were distributed over the 5-year life of the exoskeleton. 

Having at disposal all the technical data regarding AGREE’s components, it was possible to create a bill of materials. The calculation of the environmental impact also took into account the electricity consumption, as described above, together with the accompaniment of patients carried out by caregivers. 

Regarding the disposal and recycling processes, two main processes were considered as reference for the disposal of mechatronic devices: manual disassembly and shredding/mechanical separation. Then, every component of AGREE was considered either disassembled manually, or shredded/separated mechanically. At the end of these processes, the raw materials can be recycled or incinerated, generating electricity. 

In terms of recycled materials, the recyclability percentage is associated with an avoided impact, i.e., the production of new material that can be avoided by using recycled materials. 

To perform the impact assessment of LCA, the authors chose to analyse the impact category “climate change”, and the IPCC 2013 was used as a characterization model. As a result of LCA, the authors obtained the emissions caused by all the lifecycle processes, in terms of CO_2_ equivalents: they were equal to 2233.09 kgCO_2_e/year.

In order to monetize these CO_2_ emissions, it was considered a cost of 0.116 EUR/kgCO_2_e (https://www.ecocostsvalue.com/eco-costs/, accessed on 10 February 2023). 

The final value of the AGREE environmental impact is equal to 259.04 EUR/year, where the most relevant phase in terms of environmental contribution is the use phase. 

### 3.5. SROI Computation

Having calculated all the economic, social and environmental outcomes, it was possible to calculate the final SROI. In particular, four values of the SROI will be reported: the SROI computed for one exoskeleton, the SROI computed for the number of exoskeletons projected to be sold, and both with and without the environmental impact. The SROI values are shown in Table 6.

### 3.6. Sensitivity Analysis

For carrying out the sensitivity analysis, the SROI was calculated by varying two parameters. The first one, which was particularly relevant for the calculation, is the QoL. In this study, the increase in QoL of 19% was considered equal for robotic and traditional rehabilitation. 

Of course, by varying this assumption, results change; the authors decided to vary the QoL after rehabilitation of ±5 percentage points (pp), to see how the results could change. 

Improving the QoL of 14%, instead of 19%, the results are reported in the Table 7. 

Improving the QoL of 24%, instead of 19%, the results are reported in Table 8.

The second parameter that was changed for conducting the sensitivity analysis was the discount rate, to verify the extent to which the social validity of AGREE would change in relation to changes in the discount rate. To do this, we decided to increase the discount rate from 3.77% to 5%, and the results are reported in the Table 9.

## 4. Discussion

This study sought to develop a model to assess the social and environmental impact of healthcare technologies, with a focus on an exoskeleton for upper limb rehabilitation developed by a team of researchers at Politecnico di Milano. We decided to employ the SROI methodology, being able to embrace various typologies of impact, and to also include in the SROI calculations an LCA analysis for assessing the environmental impact of the considered technology.

The final SROI value resulted largely over 1:1 for all the considered cases, meaning that AGREE can be considered worthy of investment. 

The first thing that can be pointed out is the fact that considering the forecast sales the index decreases with respect to the SROI calculated for the single exoskeleton. This can be explained by the fact that outcomes are calculated in a cumulative way: by increasing the number of exoskeletons sold during the years, the outcomes increase but so too do the inputs associated with the purchase and use of these exoskeletons. However, the SROI calculated for multiple exoskeletons still remains high. 

Another important aspect to be analysed is the low relevance of the inclusion of the environmental assessment in the SROI computation. Indeed, this is connected to the fact that a rehabilitation exoskeleton is largely not environmentally damaging technology, while, as evident from the results, it is able to create a great social impact. However, the authors have contributed to provide an indication of how the environmental impact can be included in the SROI calculations. Since for other types of technologies or drugs it might give very different results, it should be a good practice to consider it in these evaluations.

It should also be taken into account that, although the sum of benefits exceeds the sum of costs, the distribution of benefits and costs may differ between different stakeholders. In particular, patients have more benefits than other stakeholders, even if they do not pay for their rehabilitation, which is guaranteed by the universal public health care system. The SROI methodology does not take into account this difference in the nature of benefits and costs. However, an increase in benefits for patients is reflected in an increase in benefits for their caregivers, their employers, and, thus, for the society as a whole. Thus, patients are not the payers of their rehabilitation, but this should not be an obstacle to the adoption of this technology, since their improvement represents a long-term benefit to society as a whole. 

Finally, the authors conducted the sensitivity analysis by increasing or decreasing the improvement of QoL by 5 pp. This is because two scenarios can be taken into account. 

Firstly, the QoL of patients treated with AGREE results higher than the QoL of patients treated with traditional rehabilitation. In this case, the SROI would be much higher, reaching a value around 4.6:1 for the single exoskeleton and 3.5:1 for multiple exoskel-etons. 

Second, another possibility could be that robotic rehabilitation, contrary to what experts and authors assumed, may have worse outcomes than traditional rehabilitation. This would be a negative result for this study, but, as the sensitivity analysis reported, the SROI would always remain higher than 1. 

Additionally, performing the sensitivity analysis by varying the discount rate from 3.77% to 5%, the SROI remained largely higher than 1, proving that the value of this technology would remain high even considering variations in the discount rate.

This paper contributes to research both from a theoretical and a practical point of view. This paper provides a model that merges the SROI methodology with the LCA. Even if the results were not particularly different considering or not the LCA analysis, other results could be obtained if the model were applied to other technologies. Moreover, this research enlarges the number of SROI studies applied to the healthcare field. 

From a practitioner’s point of view, this model could be used by decision-makers for assessing the value of robotic rehabilitation. Assumptions can be varied from case to case, but it offers a reference framework for conducting this type of evaluation. 

Finally, this investigation has also some limitations: first of all, subjectivity. Subjectivity is typical of SROI calculations, but may also be related to the fact that AGREE is still in development, and therefore, only preliminary information is available. Subjectivity can be solved through stakeholder involvement, and expert opinion elicitation contributed in this sense. However, it would be greatly useful also to interview patients and caregivers, and this can be a future research direction. Subjectivity is inherent also in the LCA analysis, since the choice of the impact category and of the monetization method can differ from analyst to analyst. 

In addition, the model is based on assumptions, and is therefore subject to uncertainty. The number of patients tackled by the technology is based on a strong assumption, as well as the QoL is based on a previous study but it could vary. In the future, these values could be refined, by interviewing all the stakeholders involved in the process and precisely evaluating all inputs and outcomes.

## 5. Conclusions

The results obtained from the application of this model satisfied the research objective of this research, and the AGREE technology resulted in being value for money.

This research provides the application of a model to a specific technology; however, it could also be generalized and employed for evaluating other kinds of technologies. The SROI analysis returns a number that is easy to be comprehended and compared to other cases. 

This study was not limited to assessing the economic value of this biomedical technology, as cost–benefit analyses might do. Rather, it attempted to evaluate the economic, social and environmental consequences of one healthcare technology, resulting in a model that could be used in decision-making processes. The SROI, coupled with the LCA, proved to be a good method to assess the extensive impact of a technology on society as a whole.

Future developments of this research include the possibility of involving more stakeholders in the analysis, in order to broaden the perspectives and reduce the uncertainty of the study. 

## Figures and Tables

**Table 1:** Patients’ social value chain.

Input	Output	Outcome	Impact
Working hours dedicated to performing the rehabilitation	Patients’ rehabilitation	Better health of patients treated with the technology (QoL)	Social reintegration
		Reduced therapy time	

**Table 2:** Hospitals’ social value chain.

Input	Output	Outcome	Impact
Exoskeletons purchased	Patients’ rehabilitation	Increase in number of treated patients	Increase the prestige of the hospital as a rehabilitative centre
Exoskeleton energy consumption			

**Table 3:** Physiotherapists’ social value chain.

Input	Output	Outcome	Impact
Workforce	Patients’ rehabilitation	Increase in knowledge about robotic technologies for rehabilitation Saved time to perform therapies with other patients	Careers enhancement

**Table 4:** Caregivers’ social value chain.

Input	Output	Outcome	Impact
Time lost waiting for the patient during the rehabilitation activity Transportation costs	Patients’ rehabilitation	Increase in free time	Social and work reintegration

**Table 5:** Employers’ social value chain.

Input	Output	Outcome	Impact
/	Patients’ rehabilitation	Reintegration of patients in the workplace	Higher productivity for the company

**Table 6:** SROI values.

SROI for a Single Exoskeleton	3.76:1
SROI for multiple exoskeletons	2.869:1
SROI + LCA for a single exoskeleton	3.75:1
SROI + LCA for multiple exoskeletons	2.868:1

**Table 7:** Sensitivity analysis QoL − 5 pp.

SROI for a Single Exoskeleton	2.882:1
SROI for multiple exoskeletons	2.199:1
SROI + LCA for a single exoskeleton	2.88:1
SROI+ LCA for multiple exoskeletons	2.198:1

**Table 8:** Sensitivity analysis QoL + 5 pp.

SROI for a Single Exoskeleton	4.697:1
SROI for multiple exoskeletons	3.584:1
SROI + LCA for a single exoskeleton	4.695:1
SROI+ LCA for multiple exoskeletons	3.583:1

**Table 9:** Sensitivity analysis with discount rate 5%.

SROI for a Single Exoskeleton	3.633:1
SROI for multiple exoskeletons	2.740:1
SROI + LCA for a single exoskeleton	3.631:1
SROI+ LCA for multiple exoskeletons	2.739:1

## Data Availability

No new data were created and all relevant data are contained within the article.
